# A novel framework based on deep learning for COVID-19 diagnosis from X-ray images

**DOI:** 10.7717/peerj-cs.1375

**Published:** 2023-06-06

**Authors:** SeyyedMohammad JavadiMoghaddam

**Affiliations:** Computer Engineering, Bozorgmehr University of Qaenat, Qaen, South Khorasan, Iran

**Keywords:** DenseNet, Deep learning model, COVID-19 detection, Loss function, X-ray images

## Abstract

**Background:**

The coronavirus infection has endangered human health because of the high speed of the outbreak. A rapid and accurate diagnosis of the infection is essential to avoid further spread. Due to the cost of diagnostic kits and the availability of radiology equipment in most parts of the world, the COVID-19 detection method using X-ray images is still used in underprivileged countries. However, they are challenging due to being prone to human error, time-consuming, and demanding. The success of deep learning (DL) in automatic COVID-19 diagnosis systems has necessitated a detection system using these techniques. The most critical challenge in using deep learning techniques in diagnosing COVID-19 is accuracy because it plays an essential role in controlling the spread of the disease.

**Methods:**

This article presents a new framework for detecting COVID-19 using X-ray images. The model uses a modified version of DenseNet-121 for the network layer, an image data loader to separate images in batches, a loss function to reduce the prediction error, and a weighted random sampler to balance the training phase. Finally, an optimizer changes the attributes of the neural networks.

**Results:**

Extensive experiments using different types of pneumonia expresses satisfactory diagnosis performance with an accuracy of 99.81%.

**Conclusion:**

This work aims to design a new deep neural network for highly accurate online recognition of medical images. The evaluation results show that the proposed framework can be considered an auxiliary device to help radiologists accurately confirm initial screening.

## Introduction

In late 2019, an event surprised the world. A new coronavirus spread rapidly to many countries and was announced as a pandemic. This virus infection is termed COVID-19. Moreover, the number of patients reached 761 million, and 6.8 million died by 26 March 2023 (WHO).

Early detection of positive cases is an impressive method to control the outbreak rate (Abdulkareem et al., 2022). The conventional way to detect COVID-19 is a real-time reverse transcription-polymerase chain reaction (RT-PCR). However, the low availability of diagnostic kits and the low accuracy of the test have prompted researchers to look for additional diagnostic tools like using computed tomography (CT) scans (Abd Elaziz et al., 2021; Lu et al., 2023) and X-ray images (Rajinikanth et al., 2022; Yousri et al., 2021). Since the wrong diagnosis of COVID-19 can cause more spread of this disease, the accuracy of diagnosis plays a vital role in controlling the disease.

Hasoon et al. (2021) proposed a method for the detection of COVID-19 that combines feature selection operator and classifiers. The accuracy of this model was 98.66%.

Many researchers have shown that the convolutional neural network (CNN) is suitable for diagnosing COVID-19 cases (Liu et al., 2022; Narin, Kaya & Pamuk, 2021). Shibly et al. (2020) proposed a model that combines a network based on a virtual geometry group and the CNN framework. The model suggested by Abraham & Nair (2020) uses multi-CNN with a feature selection based on correlation and a classifier. Researchers (Khan, Shah & Bhat, 2020) applied Xception architecture to pre-train the image datasets. All mentioned models describe COVID-19 detection as a two-classes problem. Furthermore, the maximum accuracy of the mentioned methods was 97.44.

Some studies have concentrated on combining the CNN technique with the deep learning method, such as K-nearest neighbor (KNN) (Asif et al., 2020; Ohata et al., 2020). Purohit et al. (2022) described a multi-image augmentation with the CNN network. Majeed et al. (2020) first applied a CNN-based technique to COVID-19 diagnosis. Then, they used a mapping method to adjust the parameters of the network. Mohammadi et al. (2020) performed four pre-train in the CNN-based model. The proposed model by Elaziz et al. (2020) uses a fractional multichannel that processes on a multi-core framework. Zhang et al. (2020) proposed a method based on a one-class classifier, including feature selection, infection detection, and prediction modules. The maximum accuracy of the methods which combine the CNN technique was 99.1%.

The success of DL-based approaches in automatic COVID-19 detection has necessitated a diagnosis system using these techniques (Alyasseri et al., 2022). Nagi et al. (2022) evaluated deep learning methods for COVID-19 detection using X-ray images. The best accuracy was 94.2%. Mohammed et al. (2022) introduced a novel swarm optimization method to select the optimal deep-learning model. An accuracy of 91.46% was achieved. Shoeibi et al. (2020) classified various DL models for COVID-19 into four categories.

The first one introduces the methods for classification models. These approaches present an automatic technique for a diagnosis of COVID-19 infection, including DenseNet (Sarker et al., 2020), Visual Geometry Group (VGG) network (Ardakani et al., 2020), 2D/3D CNN (Bayoudh, Hamdaoui & Mtibaa, 2020), XceptionNet (Singh, Siddhartha & Singh, 2020), NasNet mobile (Ahsan et al., 2020), AlexNet (Cortés & Sánchez, 2020), ShuffleNet (Zhang et al., 2018), SqueezeNet (Ucar & Korkmaz, 2020), GoogLeNet (Yu et al., 2020), CapsNet (Toraman, Alakus & Turkoglu, 2020), and EfficientNet (Chowdhury et al., 2020). The methods of this category consider the 2-dimensional images as input which retain and use the structural information of pixels. Since the training of CNNs needs a huge amount of data, the time complexity of the methods is high.

The second category is Generation Adversarial Network (GAN). These models train a GAN similar to a simple minimax game so that some images are created using real data (Khalifa et al., 2020). An advantage of these models is the high quality of generated data. Furthermore, they try to train like a simple minimax game in which the network creates an image similar to the real one.

Segmentation models are the third ones that use the lug region for COVID-19 detection (Abd Elaziz et al., 2021). Res2Net (Gao et al., 2019), U-Net (Saeedizadeh et al., 2020), SegNet (Badrinarayanan, Kendall & Cipolla, 2017), and Fully Convolutional Network (FCN) (Voulodimos et al., 2020) models belong to this category. These models choose a network for classification termed the encoder network in which fully connected layers are deleted. Likewise, a decoder is built to transform the low-resolution maps into the original ones.

Finally, forecasting models that use a recurrent neural network (RNN). The RNN models use sequential information and previous calculations to provide the output. In other words, a memory function in the RNN model stores previously calculated information. However, the basic RNN structure cannot be taught long-term dependencies (Jelodar et al., 2020).

This article presents a DenseNet-based architecture to help with the COVID-19 recognition system using prominent elements of X-ray images. Jaiswal et al. (2020) introduced a DenseNet201-based approach that uses a pre-train phase by considering learned weights for the ImageNet dataset. Shelke et al. (2020) introduced a classification model in which the DenseNet-161 segregates COVID-19 and normal cases. Researchers (Sarker et al., 2020) applied the DenseNet-121 structure with a transfer learning technique as an impressive approach to COVID-19 diagnosis.

This study aims to design a new deep neural network for highly accurate online recognition of medical images. This article proposes a novel framework including an image data loader, loss function, sampler, and optimizer. Moreover, the network structure combines some dense blocks with transition layers.

The rest of the article is as follows. Section 2 describes the dataset, the proposed framework, and the suggested network. In sections 3 and 4, the experiments are discussed. Finally, the conclusion of the results and discussion of the proposed model expresses in Section 5.

## Materials & Methods

### Dataset

This work used a COVID-ChestXRay dataset to evaluate the proposed framework. This dataset consisted of chest images gathered from different publications and websites. The number of images is 287, with varying types of pneumonia (severe acute respiratory syndrome (SARS), Pneumocystis and *Streptococcus* spp., COVID-19, acute respiratory distress syndrome (ARDS), and Middle East respiratory syndrome (MERS)) at the time of writing (Cohen et al., 2020). Moreover, a sub-dataset of 137 CXRs (PA) contains 29 COVID negatives and 108 COVID-positive cases (https://github.com/ieee8023/covid-chestxray-dataset).

## The proposed framework

**Figure 1:**
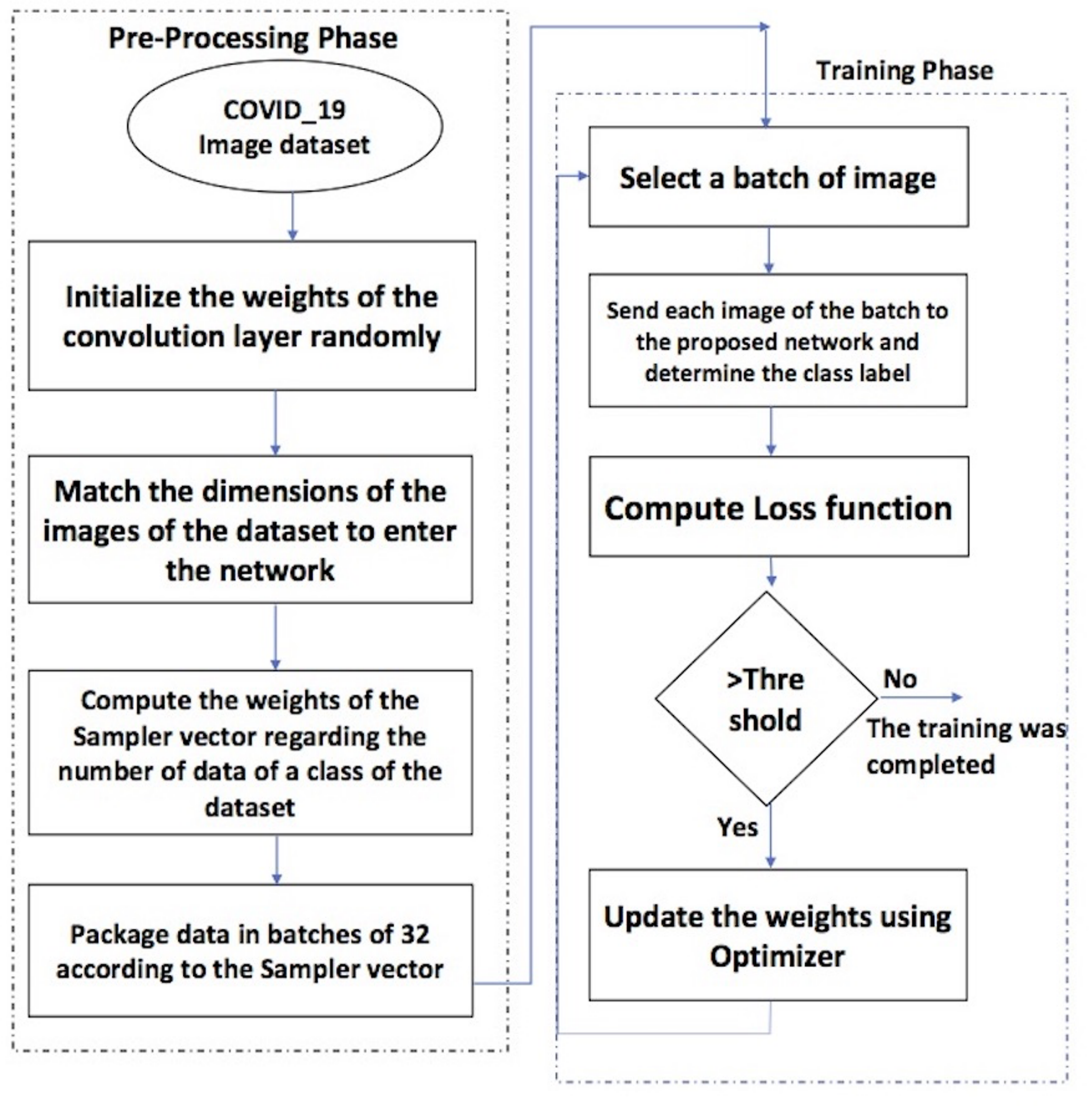
The flowchart of the proposed framework.

Figure 1 depicts the phases of the proposed framework. There are two essential phases.

The steps of the pre-processing phase are as follows.

1. The weights of the convolution layers are assigned randomly;
2. the dimensions of the images are equalized to enter the network;
3. the weights of the sampler vector have been computed according to each class’s number of data;
4. the dataset is divided into batches of 32 regarding the sampler vector

The training phase includes the following steps.

1. Select a batch of images;
2. each image of the batch is sent to the proposed network to determine the class label.
3. Sending the images of the batch to the network and determining the class label;
4. calculation of the loss function.
5. If the loss function value is more than a threshold, the optimizer updates the weights, and the next batch enters the network.
6. Repeat the above steps until the loss function value is less than the threshold

The components of the proposed framework are described in detail as follows.

### Data preprocessing

Preparing data is an important phase in solving any machine-learning problem. This step includes normalizing the images and equalizing the dimensions to enter the neural network.

### Image data loader

Preparing data is an important phase in solving any machine-learning problem. Deep neural networks need too many images to train. These images should be available in memory. Therefore, they need much memory. The image data loader is the component that provides data to models. A data loader usually (but not necessarily) takes raw information from datasets and processes them into a format the model needs. The image data loader separates images in batches; only one batch is available in each epoch of the training phase. The proposed network was trained in batch sizes 8, 16, 32, 64, and 128. The best results were achieved in batch 32.

The proposed network uses about 128 GB of RAM without an image data loader, but using it decreases the needed memory to 6 GB.

### Loss function

The machines learn with a loss function that evaluates the reasonable rate of a method that models the given data. If the prediction value diverges from real results, the loss function will return a more significant value. Gradually, the loss function learns to reduce the error in prediction using some optimization function.

Some exams have been done with various loss functions: Cross entropy, Mean Square Error (MSE), Mean Absolute Error (MAE), and Hinge Loss. Cross entropy loss originates from information theory which uses information entropy to find the magnitude of deviation between two probability distributions. It uses to train a classifier with any deep-learning framework. An ideal model has a Cross entropy of 0. Equation (1) computes Cross entropy for a binary classification setting.

(1) \begin{eqnarray*}CrossEntropy=- \left( {y}_{i}log \left( {p}_{i} \right) + \left( 1-{y}_{i} \right) log \left( 1-{p}_{i} \right) \right) \end{eqnarray*}

where *y*_i_ is the binary indicator (0 or 1) denoting the class for the sample *i* and *p*_i_ is the predicted probability between 0 and 1 for the sample.

MSE loss measures the average of the square difference between the model’s prediction and the correct values. In other words, it describes the model’s performance on the training set. MAE is similar to MSE that the absolute value of the difference instead of the square is calculated. Hinge loss is used for training classifiers in the classification of machine learning. Equation (2) calculates the hinge loss for an intended output t = ±1 and a classifier score y.

(2) \begin{eqnarray*}HLoss \left( y \right) =max \left( 0,1-t.y \right) \end{eqnarray*}

The best results were achieved using Cross-entropy.

### Sampler

The needed memory can be reduced using image data loaders. The sampler is the strategy of sampling batch images from the training dataset.

Because of the imbalanced samples in different classes of the datasets, this work uses a weighted random sampler (Efraimidis & Spirakis, 2006) to balance the training.

### Optimizer

*Optimizers* are vital components of deep neural networks that perform weight updates. *Optimizers* are algorithms or methods used to change the attributes of the neural networks, such as weights and learning rate, to reduce the losses.

Table 1 describes the training parameters of the proposed framework. The training phase assumes a batch size of 32 and a learning rate of 0.01 for 50 epochs.

### The proposed network

**Figure 2:**
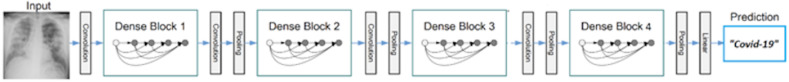
The proposed network uses a block diagram with four dense blocks.

Figure 2 (Dodiya et al., 2021) depicts the dense net diagram that the proposed network uses.

The network structure includes three transition layers and four dense blocks. The properties of the dense block and transition layer are described in the following.

### Dense block

**Figure 3:**
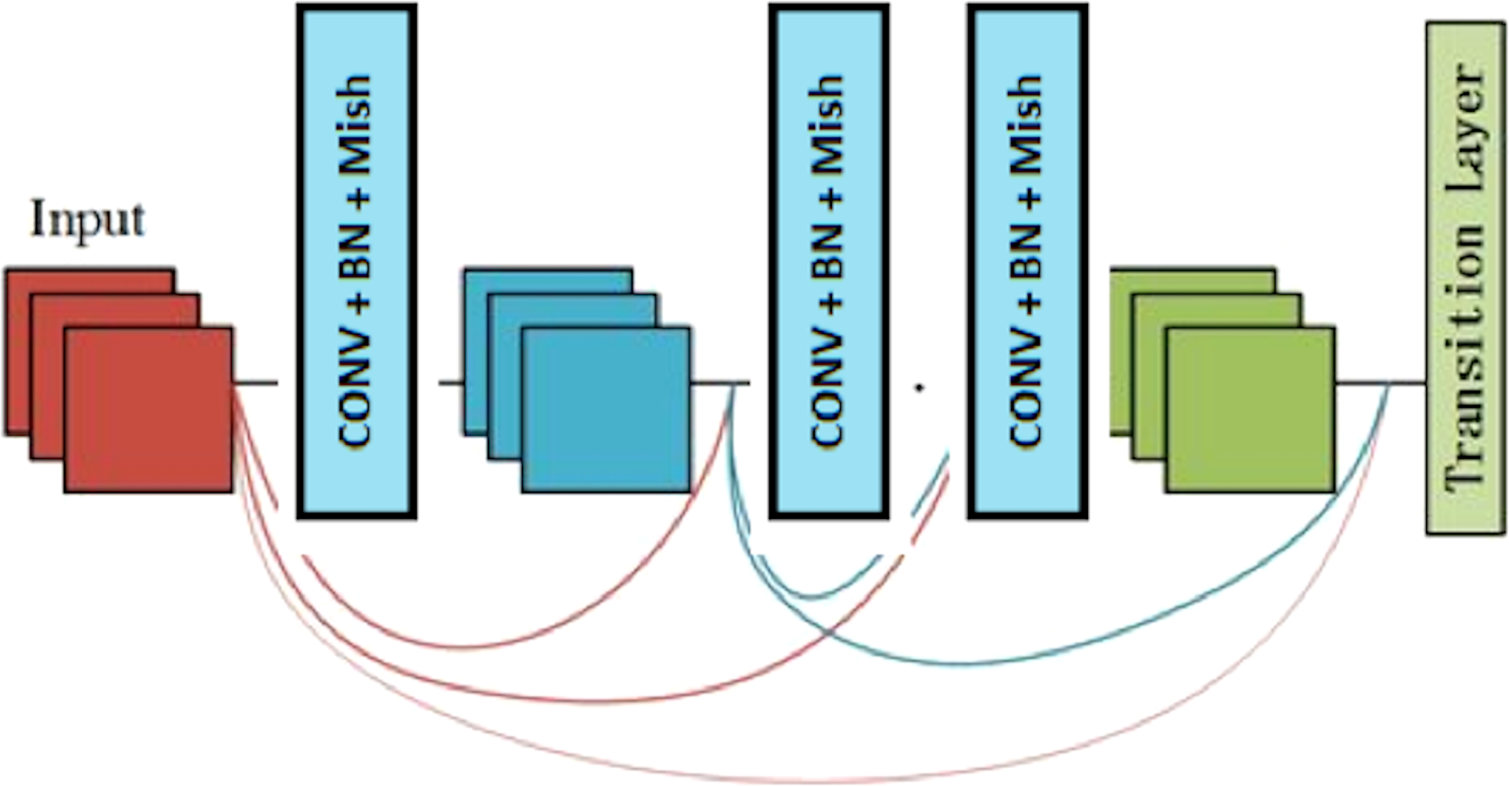
The components of the dense block module.

A dense block is a module used in convolutional neural networks that connects all layers (with matching feature-map sizes) directly with each other. A dense block comprises multiple convolution blocks with the same output channels. Figure 3 shows the structure of the dense block.

### Transition layer

It is noticeable that the dense block increases the number of channels. Therefore, adding many of them will result in a very complex model. This complexity is controlled by the transition layer, reducing the channel count by using the 1×1 convolutional layer. Moreover, the width and height of the average pooling layer are divided by a stride of 2 to reduce the complexity of the model. This work uses batch normalization (BN) (Ioffe & Szegedy, 2015) for shortening the convergence time and achieving better performance, and the Mish Misra (2019) activation function to improve the classification capacity.

Table 2 describes the components of the proposed model.

**Table 1:** Training parameters of the proposed model.

Batch size	32
Epochs	50
Weight decay	1e−3
Momentum	0.9
Epsilon	1e−10
Sampler	Weighted random sampler
Learning rate	0.01

**Table 2:** Proposed DenseNet model architecture.

Layers	Output size	Dense Block size
Convolution	112×112	7×7 conv, stride 2, padding 3
Pooling	56×56	3×3 max pool, stride
Dense Block (1)	56×56	$ \left[ \begin{array}{@{}c@{}} \displaystyle 1\times 1\text{conv}\\ \displaystyle 3\times 3\text{conv} \end{array} \right] \times 6$
Transition Layer (1)	56×56	Batch Norm + Mish + conv (1×1)
	28×28	Average pool
Dense Block (2)	28×28	$ \left[ \begin{array}{@{}c@{}} \displaystyle 1\times 1\text{conv}\\ \displaystyle 3\times 3\text{conv} \end{array} \right] \times 12$
Transition Layer (2)	28×28	Batch Norm + Mish + conv(1×1)
	14×14	Average pool
Dense Block (3)	14×14	$ \left[ \begin{array}{@{}c@{}} \displaystyle 1\times 1\text{conv}\\ \displaystyle 3\times 3\text{conv} \end{array} \right] \times 24$
Transition Layer (3)	14×14	Batch Norm + Mish + conv (1×1)
	7×7	Average pool
Dense Block (4)	7×7	$ \left[ \begin{array}{@{}c@{}} \displaystyle 1\times 1\text{conv}\\ \displaystyle 3\times 3\text{conv} \end{array} \right] \times 16$
Classification Layer	1×1	7×7 global average pool
	Num of classes	Softmax

### The modified DenseNet121

The proposed method uses an improved version of the DenseNet121 architecture. The proposed structure, in two parts, has changed the previous one, including a change in network parameters and meta-parameters. Consequently, this change has improved the model’s performance for covid-19 data.

Rectified linear unit (ReLU) is a piecewise linear function that will output the input directly if it is positive. Otherwise, it will output zero. Although adding this activator function increases the volume of calculations and occupies more of the computer’s computing memory, it improves network performance. Furthermore, the ReLU function does not remove the negative part, which causes a better flow of gradients and learning more features by the network.

Mish is a non-monotonic activation function mathematically defined according to Eq. (3).

(3) \begin{eqnarray*}f \left( x \right) =xtanh \left( ln \left( 1+{e}^{x} \right) \right) \end{eqnarray*}

The evaluation results show Mish activation function tends to improve the performance of deep learning architectures better than the ReLU function.

The network parameters differ from the (ReLU) activation function and its replacement with the M activation function.

Stochastic gradient descent with momentum (SGDM) is a method that helps accelerate gradient vectors in the right direction to achieve faster converging. The SGDM method accumulates the gradient of the past steps to determine the direction instead of using only the gradient of the current step.

Rectified Adam (RAdam) is a variant of the Adam optimizer that describes a term to rectify the variance of the adaptive learning rate. The RAdam optimizer has essential characteristics such as fast convergence and high accuracy. The change made in the network meta-parameters is to change the SGDM optimizer and use RAdam.

## Results

The experiments were conducted on a PC with GeForce Turbo RTX-2080 GPU and Corei3-9100f CPU running at 4000 MHz. Pytorch and OpenCV libraries of Python 3.7 have been used to implement the proposed algorithm and model.

**Figure 4:**
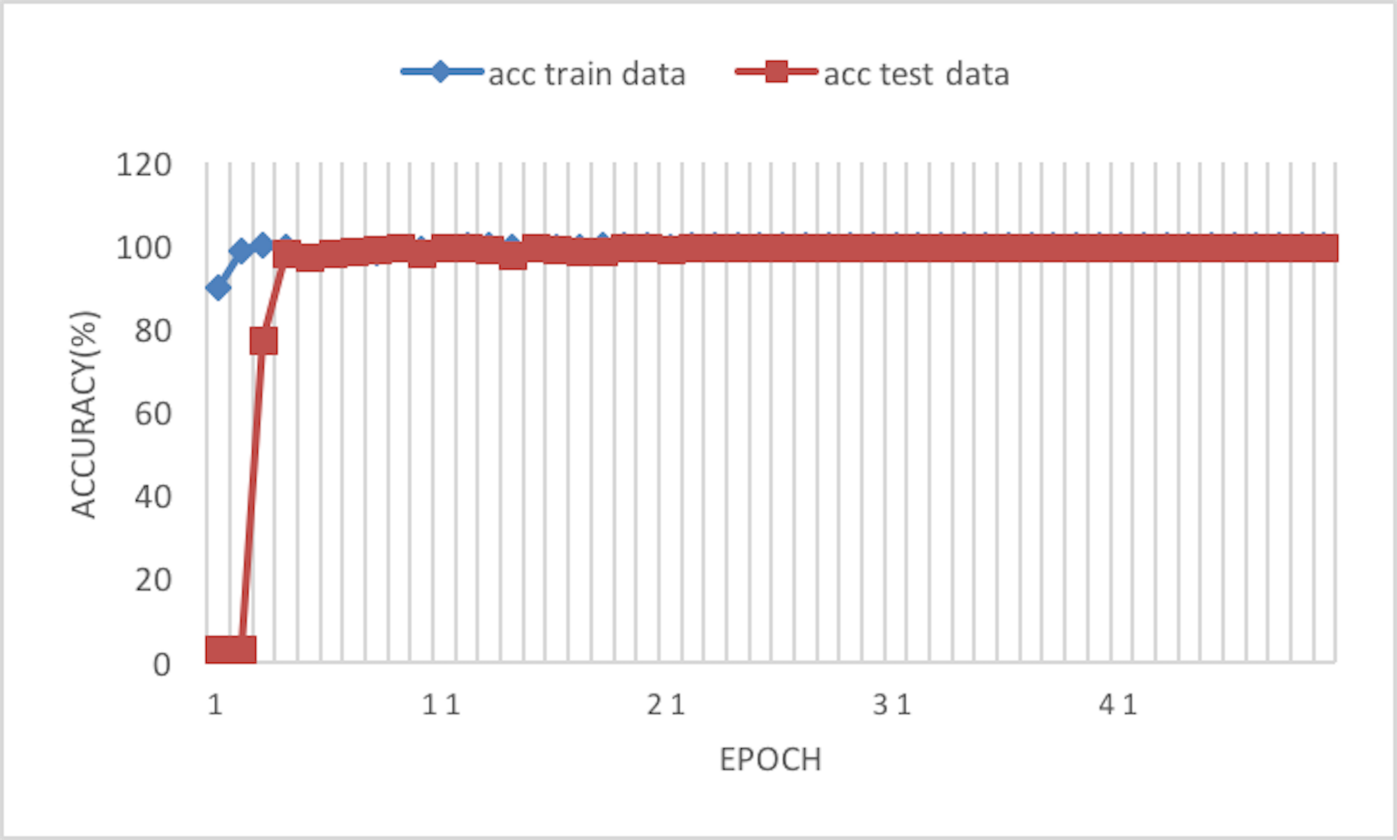
Accuracy of the network on train and test data after each epoch.

**Figure 5:**
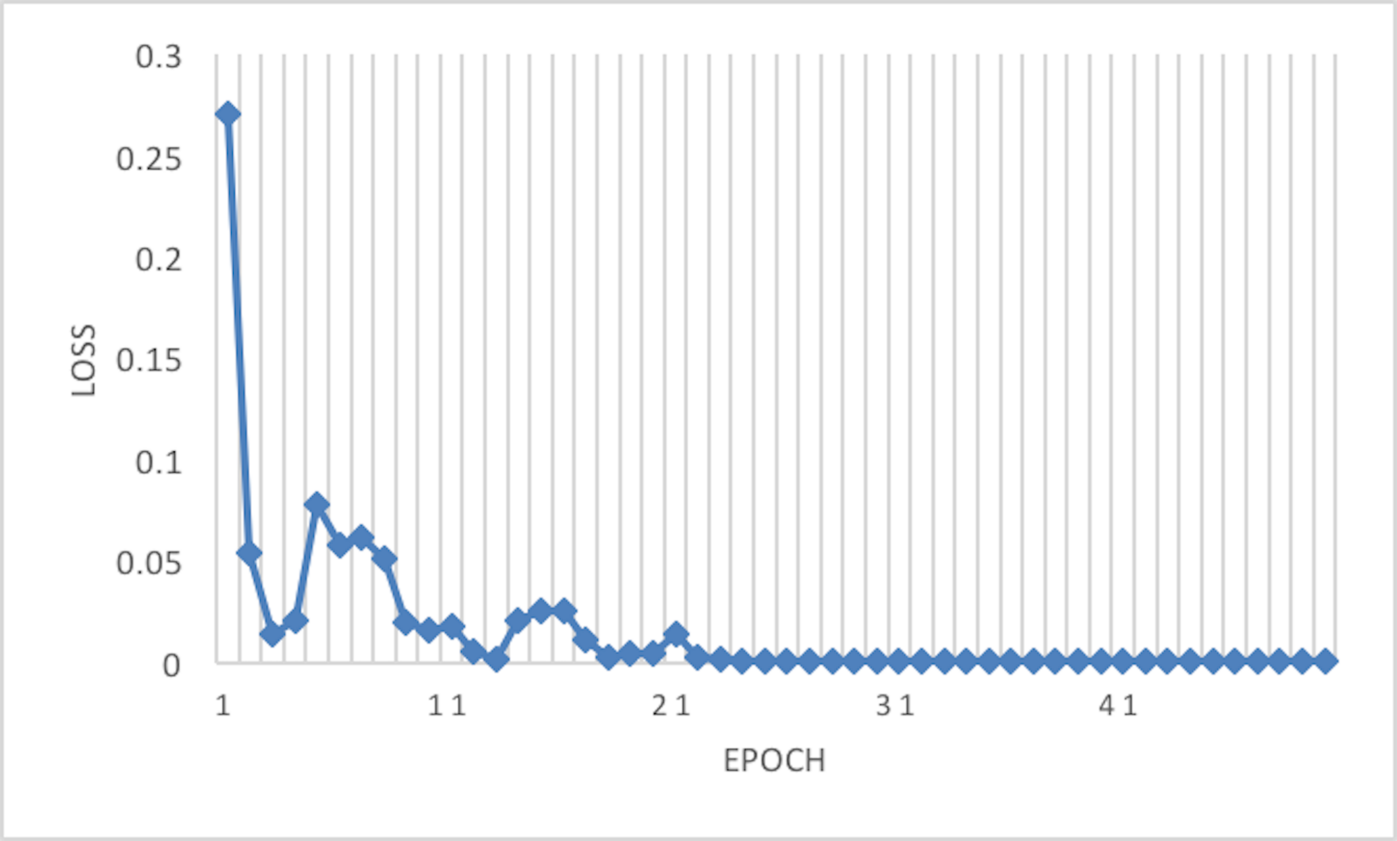
Loss of the network on train data.

The experiment phases were designing the framework, selecting the sampler, loss function, batch size, and the best optimizer. Four optimizers were used: Rectified adaptive data momentum (RAdam), whitened gradient descent RAdam (WGD-RAdam), stochastic gradient descent with momentum (SGDM), and WGD-SGDM (Gholamalinejad & Khosravi, 2022). Moreover, this study considers the image input size. Figures 4 and 5 depict each size’s accuracy and loss diagram in the best results.

The proposed model was tested in two different image sizes, 56×56 and 224×224, as Tables 3 and 4 show the results.

**Table 3:** Classification results for COVID-19 X-Ray dataset of the novel framework with input size of image 56×56.

Optimizer	Accuracy	Loss	Mean recall	Mean precision	Cohen kappa score	Recognize time
SGDM	99.55	0.0148	94.45	98.18	92.47	0.563 ms
WGD-SGDM	99.32	0.0234	94.81	94.38	89.20	0.553 ms
RAdam	99.65	0.0187	98.36	96.14	94.45	0.561 ms
WGD-RAdam	99.71	0.0126	97.43	97.89	95.32	0.573 ms

**Table 4:** Classification results for COVID-19 X-Ray dataset of the novel framework with image input size 224×224.

Optimizer	Accuracy	Loss	Mean recall	Mean precision	Cohen kappa score	Recognize time
SGDM	99.61	0.0183	97.86	96.08	93.91	0.613 ms
WGD-SGDM	98.94	0.0363	97.51	88.33	84.78	0.600 ms
RAdam	99.81	0.0122	98.93	98.80	96.92	0.565 ms
WGD-RAdam	99.39	0.0284	96.78	93.82	90.50	0.574 ms

**Figure 6:**
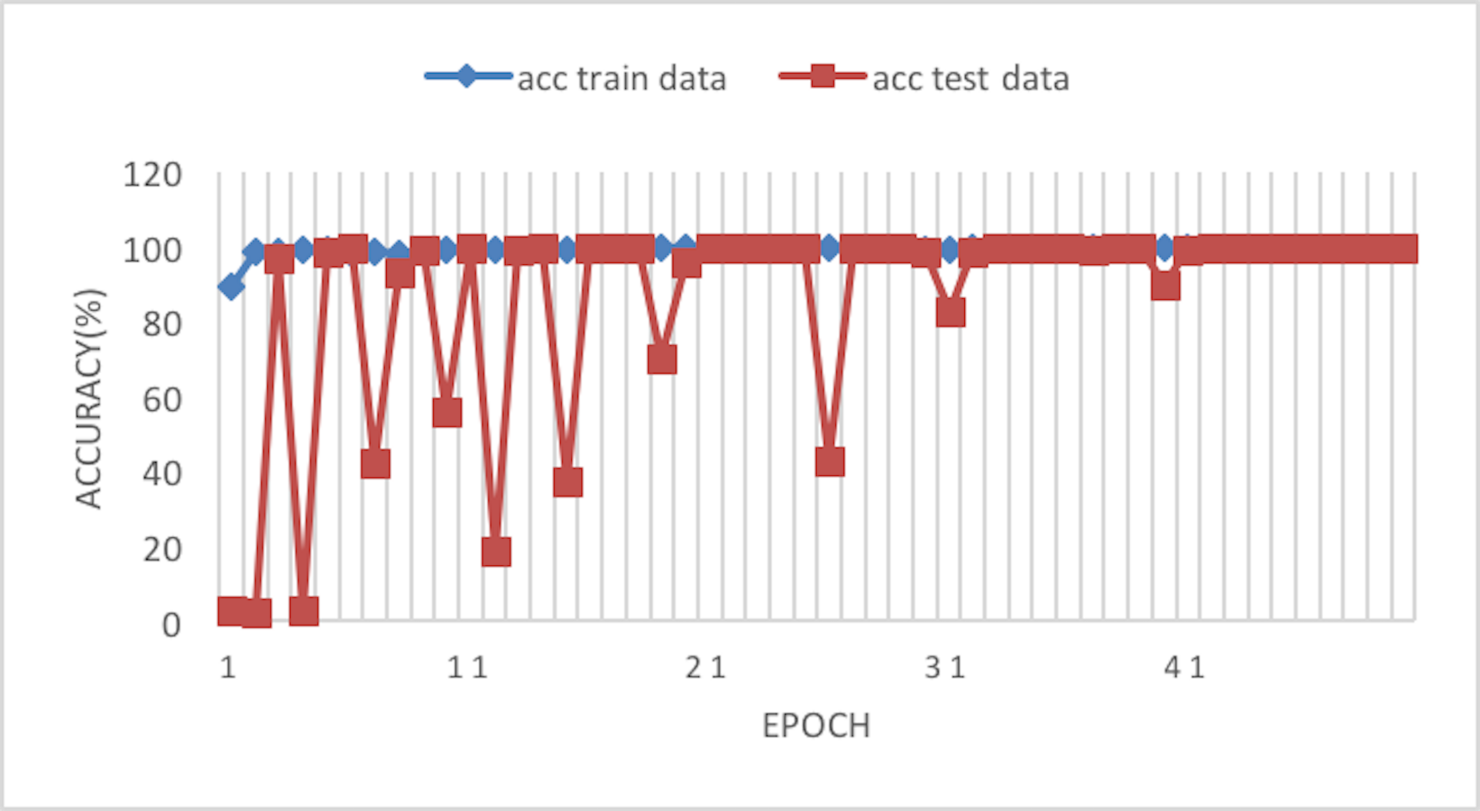
Accuracy of the network on train and test data using RAdam optimizer.

**Figure 7:**
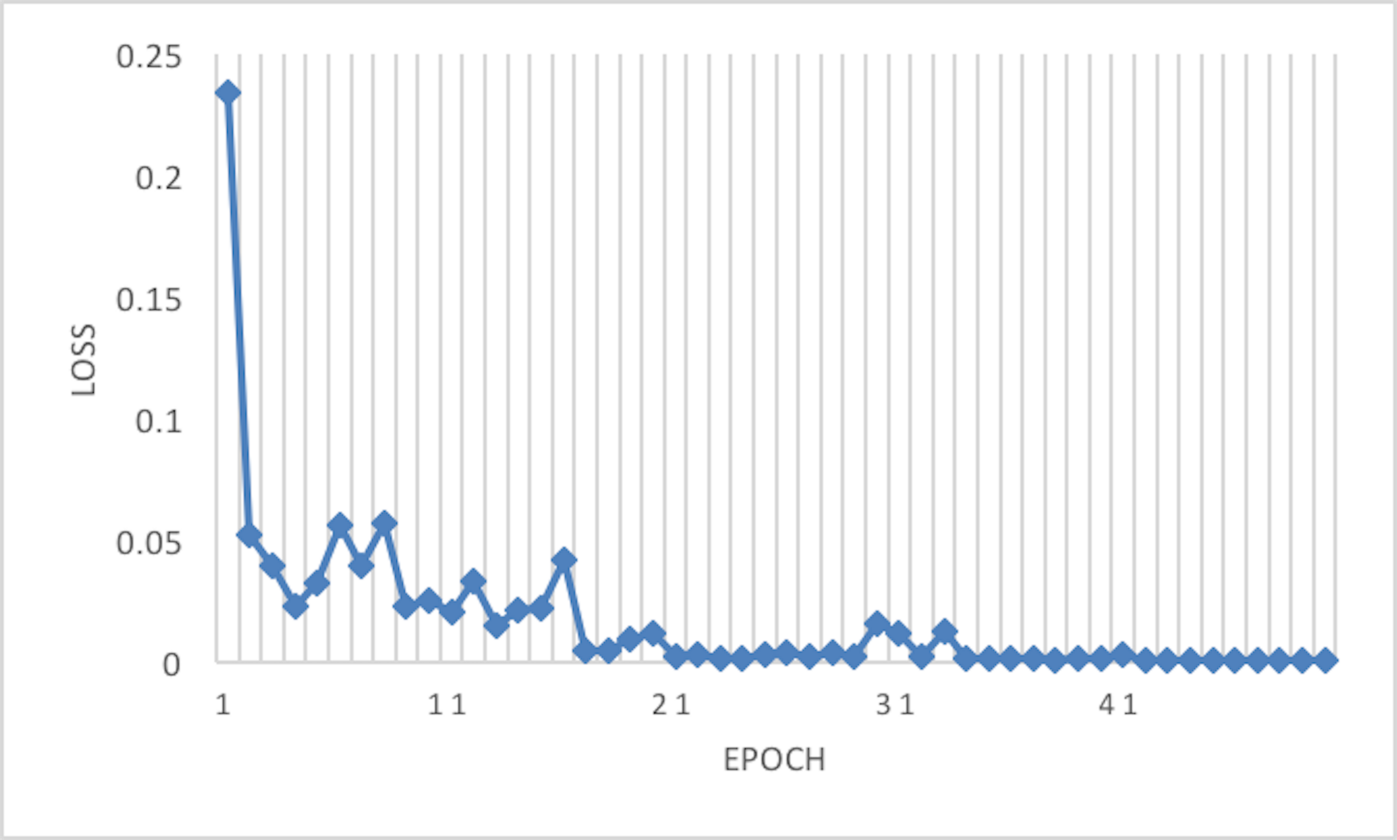
Loss of the network on train data using RAdam optimizer.

Figure 6 depicts the accuracy of the train and test data after each epoch. Moreover, Fig. 7 presents the loss curve in the training phase.

Table 5 compares the previous works based on a deep learning model for X-ray images. Since this table has been completed based on the results expressed in the text of the references, some cells are empty because the value for that classification criterion is not stated in the reference.

**Table 5:** Summary of state-of-art DL techniques used X-Ray dataset.

**Reference**	**Type of DNN**	**Dataset**	**Accuracy**	**Cohen kappa score**	**Mean precision**	**Mean recall**
Hemdan, Shouman & Karar (2020)	VGG19, DenseNet-201	Cohens GitHub	90	91	83	
Hammoudi et al. (2020)	DensenNet-169	Kaggle	95.72			
Karim et al. (2020)	DenseNet-161	3 Different COVIDx datasets	94.5	94	95
Ezzat, Hassanien & Ella (2020)	GSA-DenseNet-121-COVID-19	Combination of different datasets	98	98	98	
De Moura et al. (2020)	DenseNet-161	Combination of different datasets	99	99	100	
Sarker et al. (2020)	Modified version of DenseNet-121	COVIDx	96.4	96	96	96
Bassi & Attux (2020)	Modified version of DenseNet-121	Combination of different datasets	98.3	98.3	98.3	
Kassani et al. (2020)	DenseNet-121	Combination of different datasets	99	96	96	
Li, Li & Zhu (2020)	MobileNetv2, shuffleNetV2, Modified DenseNet121	Combination of different datasets	84.3			
Bassi & Attux (2020)	Modified version of DenseNet-201	Different datasets	99.4	99.4	99.5	
Chatterjee et al. (2020)	DenseNet161	Combination of different datasets		85.4	86.4	84.5
Proposed model	Modified version of DenseNet-121	COVID-ChestXRay	99.81	96.92	98.80	98.93

## Discussion

This article considers two parameters to evaluate the proposed method. The first one is the dimension of the input image. Another parameter is the optimizer which is used in the network training phase. According to Tables 3 and 4, the best result has been achieved using the RAdam optimizer, which is about 99.81% with a loss of 0.0122 and an image size of 224×224. Although accuracy is approximately 99.81%, the Cohen Kappa score is about 96.92% due to the mages’ complexity and the train data numbers. Figure 7 shows that after 20 epochs, the loss behavior is smooth, so the cross entropy of the network is decreased.

The suggested model overcomes the problem of model overfitting using batch normalization and adjusting the parameters of optimizers.

Since the proposed method is a kind of DenseNet network, the performance of this method has been evaluated with similar work done in the first half of 2020 with the same dataset, shown in Table 5. The point is that the experiment dataset has the lowest data according to the number. Even though there is less training data than similar research, the proposed network has given the best results according to accuracy and mean recalls. There is a relation between the Cohen Kappa score criterion and the number of training data, which is why it is less than other works.

Among the limitations of this study, the following can be mentioned. First, network performance is dependent on optimizers in neural networks. Secondly, the memory used for processing increases a lot when using Mish. Finally, the need for image datasets limits deep learning-based methods.

## Conclusions

The outbreak of COVID-19 is growing manifold daily. Due to the high speed of spread, many countries need more resources to diagnose and treat the patients. X-ray images play an essential role in diagnosing COVID-19 disease because of the expense of COVID-19 diagnosis kits and the availability of radiology equipment in most regions worldwide. Furthermore, since misdiagnosis can play an essential role in the outbreak of COVID-19, the accuracy of the diagnostic system is a critical factor. This work presents an automatic COVID-19 detection framework from the chest radiography of the patients. The model has four phases: image data loader, loss function, sampler, and optimizer. The proposed network has four dense blocks and three transition layers. Train and test phases were performed using a dataset with a few hundred X-ray images of COVID-19 and pneumonia patients. The results indicate that the framework achieved an accuracy of 99.8%. Furthermore, the RAdam optimizer achieved the best result in comparison to others. Therefore, it can be helpful to radiologists and health specialists to find out the critical aspects relevant to COVID-19 cases. Due to the smallness of the training set, the Kappa criterion of the proposed method has a lower value than other methods. In future works, according to the accuracy of the proposed model, its performance in diagnosing other diseases, such as brain tumor diagnosis, kidney stones, and balancing medical datasets, can be evaluated.

##  Supplemental Information

10.7717/peerj-cs.1375/supp-1 Supplemental Information 1 Python code

## Additional Information and Declarations

### Data Availability

The following information was supplied regarding data availability: The data is available at Github: https://github.com/ieee8023/covid-chestxray-dataset. The code is available in the Supplementary File.
